# Biomarkers in Early Diagnosis and Early Stage Lung Cancer: The Clinician’s Point of View

**DOI:** 10.3390/jcm9061790

**Published:** 2020-06-09

**Authors:** Roberto Gasparri, Giulia Sedda, Lorenzo Spaggiari

**Affiliations:** 1 Department of Thoracic Surgery, IEO, European Institute of Oncology IRCCS, Via Ripamonti, 435, 20141 Milan, Italy; giulia.sedda@ieo.it (G.S.); lorenzo.spaggiari@ieo.it (L.S.); 2 Department of Oncology and Hemato-oncology, University of Milan, 20122 Milan, Italy

**Keywords:** lung cancer, early diagnosis, biomarkers

## Abstract

Starting from the work of Ulivi and colleagues, we aim to summarize the research area of biomarkers for early diagnosis and early stage lung cancer.

It was with great interest that we read the article by Ulivi et al. [1] on the prognostic value of micro RNAs (miRNAs) as a potential prognostic marker for patients who had undergone surgery for stage I–II non-small cell lung cancer (NSCLC). Although the study was well designed, it did suffer from substantial limitations as it was lacking in validation and had little statistical strength. However, it has given us the opportunity to take stock of the “biomarker,” as seen through the eyes of a clinician focusing on “early diagnosis” and the diagnosis of molecular residual disease (MRD) [2,3] after surgery for early-stage cancer.

Lung cancer is a pandemic disease responsible for the highest rate of cancer mortality worldwide. The total number of deaths per year far exceeds the total deaths caused by the other three solid tumors, namely breast, colon, and prostate [4,5]. There are two fundamental causes of this high mortality. The first is the late diagnosis in 85% of patients (often at a stage where the cancer has become locally advanced and metastatic); the second is due to the particular biological phenotype of this cancer that affects patients who have undergone radical surgical resection. In at least 30% of such cases, survival is jeopardized [6]. As a result of this, in clinical practice, specific needs persist, such as the need to diagnose lung cancer at an early stage, as well as the need to stratify the level of risk of recurrence after surgery for an early-stage tumor, in order to prescribe adjuvant therapy if need be. In this context, the discovery of one or more molecular biomarkers could meet these needs [7] by entering the diagnostic work-up before and after a low dose chest CT scan.

As things currently stand, in practice, the low-dose chest CT scan (LDCT), a straightforward and sensitive procedure, is the only examination performed for preventive, diagnostic, and follow-up purposes for patients undergoing radical stage I surgery. In 2011, an American study [8], which constitutes a milestone in the clinical community, using LDCT revealed a reduction in mortality of 20% compared with chest X-rays in a small group of at-risk subjects (heavy smokers).

Subsequently, in 2012 [9] several clinical limitations were discussed that remain unresolved. These include the need to extend the selection criteria for population screening, the high cost of the tests performed, and the high number of false positives. For many years, progress in “precision medicine” [10,11,12] through the “omics sciences” [13,14,15] has yielded a myriad of potential biomarkers [16,17] and biological information fundamental to the discovery of lung cancer vulnerability. For many years, researchers have focused on biomarkers that could affect the physician’s strategic choice [18]. The importance of their work is inestimable. Indeed, for metastatic lung cancer, it is common standard practice to carry out liquid biopsy [12]—now an everyday reality—available to the oncologist. Biomarkers such as EGFR [19] and ALK [20] are fundamental in guiding the biological therapeutic and immunotherapeutic choice for cancer patients with adenocarcinoma (ADK) [21]. Similarly, squamous cell carcinoma in PDL-1 positive patients [22] is treated by immunotherapy.

These biomarkers bring additional crucial information to the standards of care in creating subgroups (taxa) of patients for whom the clinician will be able to set up the specific medical, biological, or immunotherapeutic treatment plan. In this context, they have created a taxonomic classification [15] of non-small cell lung cancer, based not on the tumor histology, but rather on the biological and genetic phenotype of each patient. This finding emphasizes the fact that lung cancer forms a complex and heterogeneous biological system [23,24,25,26] in which a single histology can represent several subgroups with biological microsystems that differ [27]. 

Assessing the published, peer-reviewed biomedical studies on biomarkers for early diagnosis and MRD post-surgery, one encounters an enormous amount of outstanding scientific work that reveals “signatures,” extracted from different biological fluids [28], which can potentially be employed in the clinic.

Below we will briefly highlight potential biomarkers, which have been analyzed on extraction from the blood, exhaled breath, and urine; three fluids that we believe to be clinically ideal for the choice of a future rapid, non-invasive, and scientifically robust test. 

For early diagnosis, there are several potential biomarkers. 

In the blood, autoantibodies and antigens have been evaluated [29,30,31,32], such as C4d [33]. Additionally, research has been conducted not only into miRNA [34] combined with an LDCT scan [35,36] but also into circulating tumor DNA [37,38] for which it will be essential to await the results of the Circulating Cell-free Genome Atlas Study [39]; the proteomic profile is also added to the potential signatures of early diagnosis [40,41].

In respiratory exhaled breath, an emerging research front, volatile organic compounds (VOCs), collected through simple spirometry, are being evaluated. The analysis can be performed by using gaseous mass spectrometers [42,43] to evaluate the molecular quality, and also by using artificial olfactory devices equipped with sensors that create volatile imprints, according to physical-chemical mechanisms that can differentiate healthy individuals from those with cancer [44]. It will be interesting to wait for the results of the Lung Cancer Indicator Detection (LuCID) study, which aims to validate the use of a high-throughput (www.clinicaltrials.NCT02612532) breath analysis technique.

In urine, which is abundant and easy to sample without any invasiveness, the use of liquid spectrometry [45] makes it easy to extract isolated metabolites [46] in protein panels [47]. Potentially useful for both the early diagnostic and prognostic phases [47,48,49], even though at present they are included in a discovery phase, we believe that this biological fluid can be combined with other biological fluids to create a simple, rapid and reproducible test [50]. 

Regarding post-surgery, for the diagnosis of MRD, the blood-based next generation of sequencing [51,52] (NGS) alongside comprehensive multiparameter analyses [53,54] have revealed multiple gene sequences [55,56], gene isolates [57], panels of serum proteins and autoantibody [58], panels of serum tumor antigen and autoantibodies [59], and circulating tumor cells [60]. We also note research published by E. Martinez-Terroba [61] et al. in which they identified and validated a protein-based signature, which is very promising in terms of technology used, simplicity and affordability, scientific robustness, and the potential for translation into clinical practice. Another study of notable scientific quality and simplicity is one published in 2014 by Harris [62].

The above shows the enormous dedication of researchers striving to validate specific biomarkers for both early diagnosis and post-surgery MRD. On the other hand, there is the absence of a valid and usable biomarker in the clinical context. Given the biology of lung cancer, we believe that in the future, multiple clinical-molecular-epidemiological profiles will be obtained that can identify and stratify the risk level of lung cancer. This expectation is yet to be met, due to the difficulty of translating most of the results obtained by the researchers into a clinical context. We believe in laboratory research in the clinical setting, at the patient’s bedside. It is our heartfelt hope that the individual, whether healthy or sick and who of course is central to everything, will one day be screened primarily through a non-invasive molecular test, involving sample collection of their biological fluids. The information arising from this will be combined with their medical history and epidemiological data and then possibly be followed by a low-dose CT scan.

In this regard, in our clinical institute, in both healthy patients and patients operated on for an early-stage tumor we are taking samples of blood, exhaled breath, and urine as part of a monocentric prospective study for the discovery of biomarkers. For healthy subjects, these three samples are taken in a hospital outpatient clinic; for those patients who have undergone surgery, samples are taken at the bedside. We have ascertained the logistical and technical ease of handling the three samples under the supervision of the research nurse and biologist. All the researchers involved meet regularly in our hospital and work closely with the clinicians to improve the technical aspects associated with the scientific workup of the project. 

We would like to conclude by stressing the necessity to develop projects in the future, ideally designed in clinical contexts, to translate the results from research into the application at the bedside. It will also be crucial to process the results employing standard robust statistical methods and to adhere strictly to the five steps required for the validation of each potential biomarker [63,64]. It is undoubtedly crucial to persist in the relentless search for evidence suggestive of vulnerability to lung cancer in order to weaken this ruthless killer. Each country will need to collect all the data gathered and make it transparently available in the public domain. In this way, comparisons can be drawn between data from other countries for the common purpose of creating a worldwide Big Data database [65]. Artificial intelligence [66,67] will be used to process, overlap, and integrate the molecular, clinical, and epidemiological data, and machine learning [68,69] will be employed to produce realistic multiple diagnostic algorithms which the clinician can readily use for diagnostic, preventive, and prognostic purposes. 
